# Loss of tenascin X gene function impairs injury‐induced stromal angiogenesis in mouse corneas

**DOI:** 10.1111/jcmm.13397

**Published:** 2017-11-21

**Authors:** Takayoshi Sumioka, Hiroki Iwanishi, Yuka Okada, Yuka Nidegawa, Masayasu Miyajima, Ken‐ichi Matsumoto, Shizuya Saika

**Affiliations:** ^1^ Department of Ophthalmology Wakayama Medical University School of Medicine Wakayama Japan; ^2^ Animal Center Wakayama Medical University School of Medicine Wakayama Japan; ^3^ Department of Biosignaling and Radioisotope Experiment Interdisciplinary Center for Science Research Organization for Research and Academic Information Shimane University Izumo Japan

**Keywords:** Cornea, tenascin X, neovascularization, vascular endothelial growth factor, macrophage, mouse

## Abstract

To determine the contribution by tenascin X (*Tnx*) gene expression to corneal stromal angiogenesis, the effects were determined of its loss on this response in TNX knockout (KO) mice. In parallel, the effects of such a loss were evaluated on vascular endothelial growth factor (VEGF) and transforming growth factor β1 (TGFβ1) gene and protein expression in fibroblasts and macrophages in cell culture. Histological, immunohistochemical and quantitative RT‐PCR changes determined if *Tnx* gene ablation on angiogenic gene expression, inflammatory cell infiltration and neovascularization induced by central corneal stromal cauterization. The role was determined of *Tnx* function in controlling VEGF‐A or TGFβ1 gene expression by comparing their expression levels in ocular fibroblasts and macrophages obtained from wild‐type (WT) and body‐wide *Tnx* KO mice. Tnx was up‐regulated in cauterized cornea. In Tnx KO, macrophage invasion was attenuated, VEGF‐A and its cognate receptor mRNA expression along with neovascularization were lessened in *Tnx* KOs relative to the changes occurring in their WT counterpart. Loss of *Tnx* instead up‐regulated *in vivo* mRNA expression of anti‐angiogenic VEGF‐B but not VEGF‐A. On the other hand, TGFβ1 mRNA expression declined in *Tnx* KO cultured ocular fibroblasts. Loss of *Tnx* gene expression caused VEGF‐A expression to decline in macrophages. *Tnx* gene expression contributes to promoting TGFβ1 mRNA expression in ocular fibroblasts and VEGF‐A in macrophages, macrophage invasion, up‐regulation of VEGF‐A expression and neovascularization in an injured corneal stroma. On the other hand, it suppresses anti‐angiogenic VEGF‐B mRNA expression *in vivo*.

## Introduction

Maintenance of corneal avascularity and transparency is critical to normal vision. Neovascularization in the corneal stroma induced by microbial infection or tissue injury rises leads to rises in corneal tissue in angiogenic factor expression and neovascularization. Such effects are potentially associated with corneal opacification and impair vision. Angiogenic factors mediate this phenomenon through a complex system of various growth factor signalling pathways 1, 2, 3, 4, 5. Along with these changes, corneal injury results in both inflammatory cell infiltration and resident corneal cell activation. Specifically, keratocytes and epithelial cells express angiogenic growth factors, such as platelet‐derived growth factor, VEGF, transforming growth factor β (TGFβ) and fibroblast growth factor 6, 7, 8, 9, 10, 11. The VEGF family has several members; VEGF‐A is reportedly most potent in inducing corneal neovascularization, while VEGF‐B has quite limited angiogenic activity or even is pro‐anti‐angiogenic 12, 13, 14. Its anti‐angiogenic activity is consistent with the finding that blocking VEGF‐B expression reportedly promotes neovascularization in rat corneas 15.

The stromal extracellular matrix (ECM) forms the ground substance supporting collagenous its framework in which keratocytes, fibroblasts and myofibroblasts are dispersed. Their activation by injury and/or infection induces neovascularization *via* a direct effect of angiogenic factors on vascular endothelial cell proliferation and elongation along with increases in proinflammatory cytokine release by activated resident immune cells 16, 17, 18, 19, 20, 21. Such control over these responses occurs through the same pathways activated during embryogenesis as well as in pathological conditions, *i.e* cancer and wound healing. The matricellular protein modulators contributing to inducing these responses include families of tenascins, osteopontin, periostin 22, 23, 24, 25, 26, 27, 28, 29.

Tenascins are a family of ECM matricellular proteins that consists of five members; tenascin C, R, X, Y and W. They share a unique pattern of four domains: heptad repeats, epidermal growth factor‐like repeats, fibronectin type III‐like repeats and a globular domain shared with fibrinogens 30, 31. We previously reported that injury‐induced neovascularization in corneal stroma was suppressed in a mouse line that lacks tenascin C 32.

Tnx is a 450 kD glycoprotein 33, having an important role contributing to the control of collagen fibrillogenesis in tissues 34. Mutation of the *TNX* gene in humans is causative for Ehlers‐Danlos syndrome. It was recently reported that Tnx also modulates the effects of growth factors. For example, TNX reportedly activates the latent form of endogenous TGFβ1 and accelerates epithelial‐mesenchymal transition in cell culture 35, 36. Furthermore, we reported that TNX binds to both VEGF‐A and ‐B and facilitates VEGF‐B/VEGF‐R1 receptor‐mediated proliferation of vascular endothelial cells 37, 38. In addition, it was shown that loss of Tnx gene function counteracts VEGF‐A promotion of endothelial cell proliferation in an explant of a mouse embryo in organ‐culture 38.

In this study, we determined if lacking Tnx affects expression or tissue accumulation of angiogenic factors and development of neovascularization in corneal stroma in response to injury in mice using our previously generated *Tnx* ‐null (KO) mice 39. Furthermore, we tested if loss of Tnx expression alters expression of VEGFA and VEGFB or TGFβ1 in fibroblasts and macrophages, respectively.

## Materials and methods

Each experimental protocol was approved by the DNA Recombination Experiment Committee and the Animal Care and Use Committee of Wakayama Medical University, and performed in accordance with the Association for Research in Vision and Ophthalmology Statement for the Use of Animals in Ophthalmic and Vision Research.

### Stromal neovascularization by cauterization of the central cornea in mice

C57BL/6 WT and KO mice in a C57BL/6 background 39 were used. WT and KO corneal histological findings were identical with one another (data not shown). Stromal neovascularization from the limbal vessels was induced by cauterization of the central cornea of an eye with a Optemp disposable tool 8. One eye of both WT (*n* = 45) and KO mice (*n* = 47) was used for induction of corneal neovascularization and then killed on either day 3, 7 or 14 by asphyxiation. The eye was then enucleated and processed for histology and immunohistochemistry by obtaining cryosections (WT: *n* = 36; KO: *n* = 38) and paraffin sections (WT: *n* = 9; KO: *n* = 9).

### Histology and immunohistochemistry

Because simple haematoxylin and eosin (H&E) staining histology is not suitable for detection of neovascularization in corneal stroma, we carried out CD31 antigen immunohistochemistry. Cryosections (7 μm in thickness) from all the samples were fixed in cold acetone for 5 min. and processed for immunohistochemistry with a rat monoclonal anti‐CD31 antibody as previously reported 10, 40. The sections were then observed under light microscopy. Statistical analysis of the data of the distance between CD31‐positive limbal vessels and the tip of the neovascularization in the corneal stroma was conducted by employing Tukey–Kramer's test and differences whose *P* < 0.05 were taken as significant.

VEGF‐A or ‐B (VEGF_164_ Antibody [1:100 dilution in phosphate‐buffered saline (PBS), R&D Systems, Minneapolis, MN], VEGF‐B_167/186_ Antibody (1:100 dilution in PBS; R&D Systems) and Tnx (M23 antibody) (1:50 dilution in PBS, Shimane University, Japan) were double‐stained in paraffin sections using fluorescence immunohistochemistry. F4/80 antigen (a macrophage marker) [1:100 dilution in PBS; BMA Biomedicals, August, Switzerland], myeloperoxidase (MPO, a marker for a neutrophil leucocyte) [1:100 dilution in PBS; Thermo Fisher Scientific, Fremont, CA, USA] or α‐smooth muscle actin (αSMA) [1:200 dilution in PBS, Thermo Fisher Scientific] were also immunostained in paraffin sections 3, 4. Visualization of complex of antibodies was performed by immunoperoxidase reaction with diaminobenzidine or FITC‐ or rhodamine‐conjugated secondary antibody. The stained samples were then observed under light microscopy.

### Real‐time reverse transcription‐polymerase chain reaction (RT‐PCR) in *in vivo* samples

Uninjured corneas or corneas at day 3 post‐cauterization of either WT or KO mice were used. Forty WT and 40 KO uninjured corneas, as well as 48 WT and 48 KO corneas at day 3 after treatment, were used to obtain their RNA samples. Four cornea samples were merged into one tube. Total RNA extraction and TaqMan real‐time RT‐PCR were carried out to semi‐quantify the expression level of F4/80, VEGF and αSMA. TaqMan primers for mRNAs of F4/80 (Mm00802529_ml), VEGF‐A (Mm01281447_ml), VEGF‐B (Mm00442102_ml), VEGF‐receptor1 (Flt‐1: Mm00438980_ml) and αSMA (Mm01204962_ml) were purchased from Applied Biosystems (Drive Foster City, CA, USA). Data were statistically analysed by employing anova.

### Expression of VEGF‐A and TGFβ1 in KO ocular fibroblasts

To examine the effect of the loss of endogenous Tnx on mRNA expression of TGFβ1 (Mm03024053_ml) and VEGF‐A in ocular fibroblasts, real‐time RT‐PCR was performed using reported TaqMan probes 3, 4. In brief, eye globes were obtained from post‐natal 1‐day‐old WT and KO mice after CO_2_ asphyxiation and intraocular tissues were removed. The remaining tissues in the eyeshell were minced and explanted for the outgrowth of ocular fibroblasts. After two passages in 60‐mm culture dishes, the cells were re‐seeded into 60‐mm dishes, grown to confluence and were then treated with serum‐free medium for 24 hrs. Six wells were prepared for each culture condition. The cells were processed for RNA extraction to determine with real‐time RT‐PCR VEGF‐A and TGFβ1expression levels.

### VEGF‐A expression of macrophages in cell culture

Mouse macrophages were obtained from the peritoneal cavity of WT and KO mice by induction with oyster glycogen i.p. injection as previously reported by us 9, 41, and allowed to adhere to 60‐mm plastic dishes for 24 hrs. Our preliminary investigation clearly showed that WT peritoneal macrophage expresses sufficient levels of *Tnx* mRNA (data not shown). The cells were then treated with serum‐free medium for 24 hrs followed by incubation in serum‐free medium or supplemented with 1.0 ng/ml TGFβ1 for 24 hrs. RNA was extracted from either macrophage or fibroblast cultures and real‐time RT‐PCR determined mRNAs of VEGF‐A. Data were analysed by anova.

## Results

### Expression pattern of Tnx in a mouse cornea

**Figure 1:**
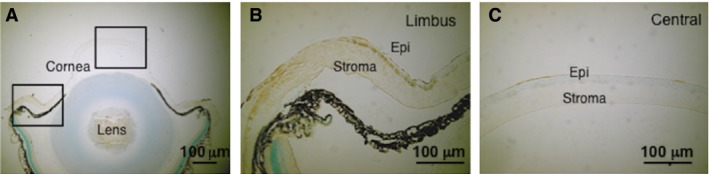
Expression pattern of tenascin X (Tnx) in a mouse cornea. (**A**) Tnx immunostaining is present in the peripheral stroma, but not in the central, cornea of an uninjured subject. (**B** and **C**) show higher magnification images of the boxed areas in **A**. (**B**) Tnx immunoreactivity is observed in epithelial basal cells and peripheral stroma of the cornea. (**C**) Central cornea lacks Tnx protein. Epi: epithelium; Stroma: corneal stroma; Scale bar is 100 μm.

Tnx immunostaining was detected in the stromal periphery and in the basal epithelial cells, whereas it was absent in the central stromal region of an uninjured cornea (Fig. 1).

### Tnx and VEGF protein colocalization pattern

**Figure 2:**
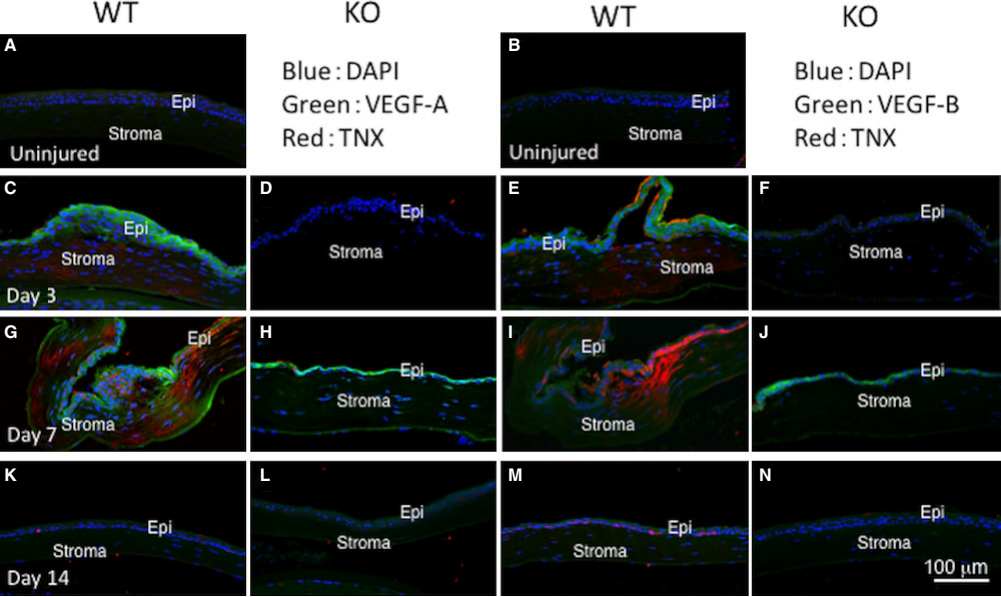
Protein expression levels of tenascin X (Tnx) and vascular endothelial growth factor (VEGF) in a centrally cauterized mouse cornea. Tnx, VEGF‐A and VEGF‐B immunostaining are present in the central part of an uninjured WT mouse cornea (**A** and **B**, respectively). At day 3 post‐cauterization, Tnx is faint, but obviously, up‐regulated in the central stroma of a WT mouse. At this time‐point, VEGF‐A and ‐B were slightly expressed in the epithelium of a WT central cornea (**C** and **E**, respectively), while immunoreactivity of these subtypes is less marked in a KO cornea (**D** and **F**, respectively). At day 7, Tnx protein is markedly up‐regulated in the stroma of the central cauterized WT cornea (**G** and **I**, respectively). At day 7, VEGF‐A protein expression is evident in the stroma of the WT central cornea as well as in the epithelium (**G**). In a KO cornea, VEGF‐A immunoreactivity is present in the epithelium, but not in the stroma (**H**). At this time‐point, VEGF‐B protein expression is only faint in the epithelium and stroma of the WT central cornea (**I**). In a KO cornea, VEGF‐B immunoreactivity is apparent in the epithelium, but not in the stroma (**J**). At day 14, there is faint Tnx expression in the corneal epithelium and stroma (**K** and **M**, respectively). VEGF subtypes are not detected in WT and KO corneas (**L** and **N**, respectively). Epi, epithelium; Stroma, corneal stroma; Bar, 100 μm.

As cauterization only induces neovascularization in the corneal periphery, we determined if this expression pattern parallels VEGF up‐regulation 31, 42. The results of double‐Tnx and VEGF immunostaining shown in Figure 2 indicate that they were undetectable in the central uninjured WT mouse cornea. At day 3 post‐cauterization, Tnx was faintly, but obviously up‐regulated in the central stroma of a WT mouse. At this time, VEGF‐A and ‐B were co‐expressed in the central epithelium, while their immunoreactivity was much less marked in a KO cornea. At day 7, Tnx protein was markedly up‐regulated in the stroma of the WT central cornea that had been cauterized. Also on day 7, there was central stromal VEGF‐A protein expression in the WT central cornea as well as in the epithelium. In a KO cornea, VEGF‐A immunoreactivity was observed in the epithelium, but not in the stroma. At this time‐point, VEGF‐B protein was detected faintly in the epithelium and stroma of the WT central cornea. In a KO cornea, VEGF‐B immunoreactivity was evident in the epithelium, but not in the stroma. At day 14, Tnx was very faint in the epithelium and stroma. Both VEGF subtypes were absent in the WT and KO uninjured corneas.

### VEGF subtype mRNA expression patterns in centrally cauterized cornea

**Figure 3:**
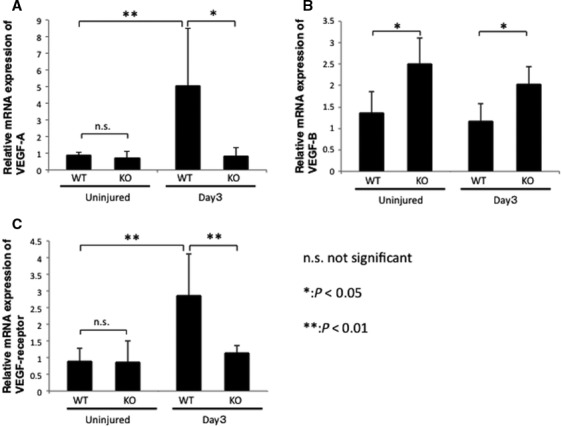
mRNA expression of angiogenic growth factors in centrally cauterized cornea. (**A**) Relative VEGF‐A mRNA expression level is significantly up‐regulated at day 3 post‐cauterization in wild‐type (WT) tissues. This up‐regulation is totally abolished by the loss of Tnx at this time‐point. (**B**) Relative expression level of VEGF‐B mRNA is not affected by the cauterization in the central cornea. Lacking Tnx up‐regulated VEGF‐B expression levels in an uninjured cornea as well as at day 3 following cauterization. (**C**) Expression level of VEGF receptor mRNAs is significantly up‐regulated at day 3 post‐cauterization in WT tissues. This up‐regulation response is totally abolished by the loss of Tnx at this time‐point. *P < 0.05; **P < 0.01 significant by anova test.

To examine whether there is a correspondence between changes in VEGF‐A and VEGF‐B in KO tissues mRNA expression and their protein expression levels in cauterized WT and *Tnx* KO mice, corresponds with less, real‐time RT‐PCR determined VEGFs and VEGF receptor mRNA expression levels in extracts of these corneas. VEGF‐A mRNA expression was significantly up‐regulated in the cauterized corneas at day 3, whereas this up‐regulation was totally abolished by the loss of *Tnx* gene function (Fig. 3A). On the other hand, VEGF‐B mRNA expression was higher in KO tissue than in both healthy, uninjured WT tissue and also at day 3 post‐cauterization (Fig 3B). Similarly, the expression level of VEGF‐receptor1 (Flt‐1) was also not significantly altered by the loss of Tnx in an uninjured cornea (Fig. 3C). On the other hand, its expression was up‐regulated at day 3 post‐cauterization in a WT cornea, but not in a KO cornea (Fig. 3C).

### Neovascularization in corneal stroma

**Figure 4:**
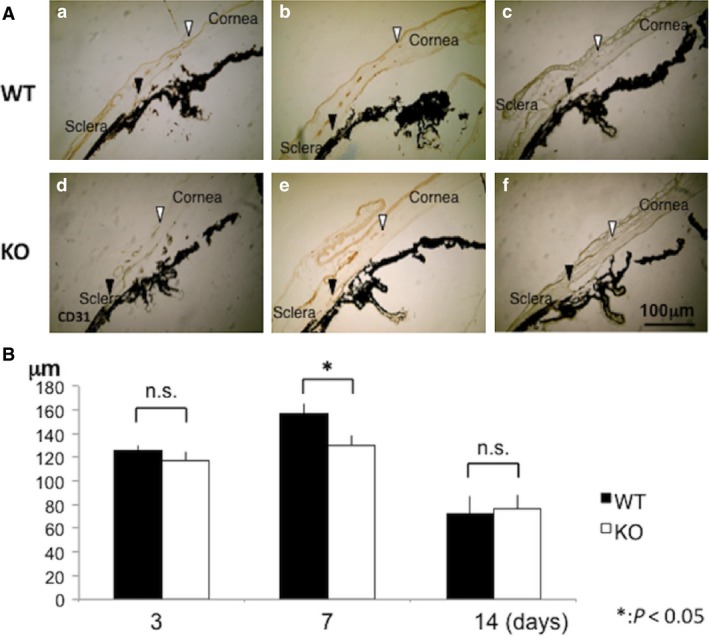
Neovascularization in peripheral stroma in response to cauterization in the central area of the cornea. (**A**) Effects of cauterization on neovascularization in corneal stroma. In WT mouse corneas, formation of CD31‐labelled neovascularization from the limbus in the corneal stroma is evident in the peripheral cornea at day 3 (Fig. 4, between black and white arrowheads). The length of such neovascularization in the corneal stroma exhibited its peak at day 7 and then started to decline at day14. (**B**) The length of the neovascularization in KO mice as compared with WT mice. (a, b and c) findings of WT mice; (d, e and f) corneas of KO mice; a and d: at day 3; b and e: at Day 7; c and f: at Day 14. Black arrow heads: point to the corneol‐limbal border; white arrow heads: point to the leading tip of the neovascularization. Scale bar, 100 μm. (**B**) Length of the stromal neovascularization as indicated by the distance between limbus (black arrows in Fig 4a) and the leading tip of the neovascularization (white arrows in Fig 4A) at each time‐point. The length of stromal neovascularization is significantly shorter in KO mice as compared with WT mice at day 7. *P <* *0.05.

As cauterization‐induced VEGF‐A or ‐B up‐regulation is associated with *Tnx* gene expression, we then examined if loss of *Tnx* gene function alters stromal neovascularization by monitoring CD31‐immunostaining in cryosections. In WT mouse corneas, CD31‐labelled neovascularization was detected extending from the limbus towards the corneal stroma from the periphery at day 3 (Fig. 4A, between black and white arrowheads). The length of such neovascularization in the stroma reached a peak at day 7 and then began to recede at day 14. The length of the neovascularization was less in KO mice as compared with WT mice at day 7, but not at days 3 and 14 (Fig. 4B).

### Inflammatory cell invasion and myofibroblast appearance in centrally cauterized cornea

**Figure 5:**
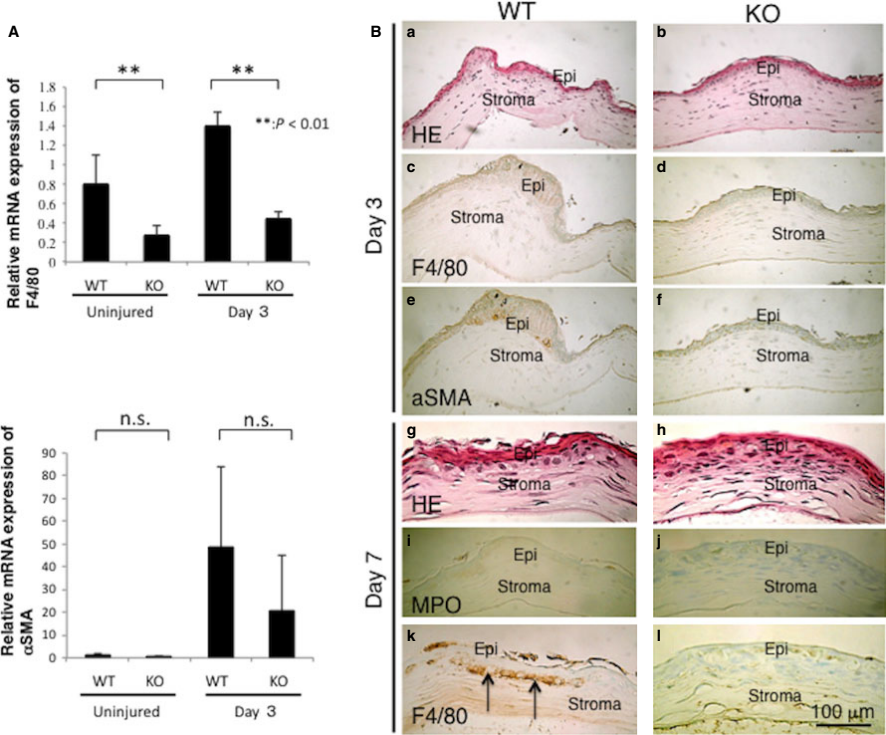
Inflammatory cell infiltration and myofibroblast appearance in centrally cauterized cornea. (**A**) Expression level of mRNA of F4/80 is significantly less in a KO cornea as compared with a WT cornea before being injured and at day 3 post‐injury. Expression level of mRNA of αSMA is not significantly altered by loss of Tnx.**P < 0.01. (**B**) Cellular distribution in the central stroma of WT and KO mice is revealed based on H&E staining at day 3 and day 7 (a, b, g, h). Macrophages were not observed in centrally cauterized corneas at day 3 (c), while at day 7 macrophages they are present beneath the epithelium in central stroma of the WT cauterized central cornea (k, arrows) (F4/80 immunohistochemistry). F4/80‐labelled cells were very few in the stroma of a KO mouse (d, l). At day 3, myofibroblasts are not present in the central stroma in both WT and KO mice (e, f). At day 7, infiltration of myeloperoxidase (MPO)‐immunolabelled neutrophils is not present in the central stroma in both WT and KO mice (i, j). Epi, epithelium; Stroma, corneal stroma; Bar, 100 μm.

We then examined if suppression of VEGF expression in the cauterized KO corneas is associated with an alteration of macrophage infiltration and keratocyte transdifferentiation into myofibroblasts. For this purpose, we examined the expression levels of F4/80, a macrophage marker and αSMA, a myofibroblast marker, in the tissue at both the mRNA and protein level. To quantify the macrophage invasion and myofibroblast expression, RT‐PCR analysis evaluated F4/80 and αSMA mRNA expression levels. Only F4/80 gene expression declined in uninjured KO mice and at day 3 post‐injury (Fig. 5A).

Immunohistochemistry was performed to identify differences in the specific cell type responses to cauterization in the WT and KO corneas. F4/80‐labelled macrophages were not observed in the centrally cauterized corneas of both WT and KO mice at day 3 (Fig. 5 B[c, d]), while at day 7 F4/80‐positive cells were observed beneath the epithelium in the WT central stroma in the cauterized area (Fig. 5 B[k]). At the same time, F4/80‐positive cells were very few in the stroma of a KO mouse (Fig. 5 B[l]). At day 3, the appearance of myofibroblasts was not detectable in WT and KO central stroma (Fig. 5 B[e, f]). At day 7, there was no central stromal infiltration of MPO‐labelled neutrophils in Tnx KO mice (Fig. 5 B[i, j]). These results suggest that lacking *Tnx* gene expression attenuated recruitment of macrophages in the cauterized corneal stroma.

### Differential effects of loss of Tnx on angiogenic cytokine mediators in fibroblasts and macrophages

**Figure 6:**
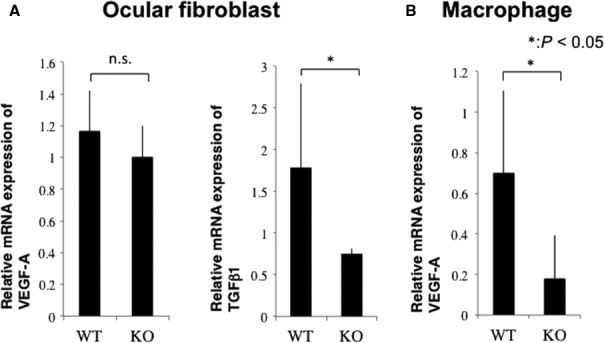
Effects of lacking tenascin X (Tnx) on angiogenic cytokine gene expression s in fibroblasts and macrophages *in vitro*. (**A**) VEGF‐A mRNA expression level is unaffected by loss of Tnx gene function in cultured mouse ocular fibroblasts. TGFβ1 gene expression level is significantly higher in WT than in Tnx KO cells.*P < 0.05. (**B**) VEGF‐A mRNA is significantly higher in WT than in Tnx KO peritoneal macrophages.*P < 0.05.

Loss of endogenous Tnx did not affect ocular fibroblast VEGF‐A mRNA expression. However, its loss significantly reduced fibroblast TGFβ1 mRNA expression (Fig. 6A). On the other hand, WT macrophage VEGF‐A expression was significantly higher than in KO cells (Fig. 6B).

## Discussion

We show here that cauterization up‐regulated Tnx expression in a WT mouse cornea, whereas loss of *Tnx* gene function attenuated stromal neovascularization induced by cauterization. Similarly, loss of Tnx gene function suppressed macrophage invasion as determined by declines in mRNA expression of the macrophage antigen, F4/80, level as well as by less VEGF‐A immunohistochemical staining. Corneal neovascularization reportedly depends predominantly on increases in the activity of VEGF‐A 12, 13, 14. Real‐time RT‐PCR also detected a significant suppression of VEGF‐A mRNA up‐regulation in the centrally cauterized mouse cornea at day 3 in the Tnx KO. In contrast, double‐immunofluorescence staining showed parallel increases in Tnx and VEGF‐A expression in the cauterized WT stroma with a peak at day 7, but not at 14, although an uninjured cornea lacks Tnx and VEGF subtype expression. VEGF receptor mRNA expression of was also up‐regulated at day 3 post‐injury in a WT cornea, whereas loss of *Tnx* gene function blocked such an effect. The finding that VEGF‐A was up‐regulated is consistent with increases in vessel formation because stromal VEGF‐A deposition having initially risen almost disappeared at day 14 post‐cauterization along with a decline in neovascularization from its peak reached at day 7. On the other hand, mRNA expression of VEGF‐B was enhanced by the loss of *Tnx* gene function in uninjured corneas, and its level remained invariant following cauterization. Although protein expression of VEGF‐B seemed more marked in the WT tissue at day 3, VEGF‐B staining in the epithelium seemed more intense in a KO tissue at day 7. Nevertheless, the immunohistochemical staining pattern of both VEGF‐A and ‐B detection agrees with other studies showing that Tnx binds to both VEGF‐A and VEGF‐B 37, 38. In the current study, the attenuation of neovascularization is considered to be attributable to decreased deposition of VEGF‐A and presumably to increased expression of VEGF‐B following cauterization 12, 13, 14, 15.

Macrophages are known to be one of the major components in an injured tissue that express angiogenic cytokines/growth factors including VEGF subtypes, although neutrophils and local mesenchymal cells also play significant roles. H&E histology identified significant levels of various different cell types in the stroma at days 3 and 7 in both WT and KO tissues. Thus, we performed immunohistochemical analysis to identify neutrophil, macrophage and myofibroblast populations. The results showed that the loss of Tnx inhibited distribution of macrophages, but neutrophils invasion and myofibroblast transdifferentiation from keratocytes were not affected by *Tnx* gene KO. This finding suggests that the decline in macrophage density might be attributable to lower tissue VEGF levels. However, these data cannot account for why the loss of *Tnx* gene function reduced both macrophage recruitment, and angiogenic and inflammatory factors expression by localized mesenchymal cells and macrophages. These effects may be instead attributable to declines in TGFβ1 expression by mesenchymal cells, which is consistent with attenuated macrophage recruitment into the tissue.

To clarify the underlying cellular pathobiological mechanisms *in vivo*, we determined if the loss of Tnx gene function affected the expression levels of angiogenic factors by fibroblasts or macrophages. Our previous study showed that endogenous Tnx C expression is required for angiogenic gene expression in cultured ocular fibroblasts, as well as development of neovascularization in a mouse corneal stroma 32. However, in the current study, the loss of *Tnx* gene function did not affect the expression level of VEGF‐A, although its expression level was up‐regulated *in vivo* in a cauterized mouse corneal stroma. Abnormal up‐regulation of TGFβ2/3 expression in patients with TNX‐related Ehlers‐Danlos syndrome phenotypes plus congenital adrenal hyperplasia was reported. TGFβ1 expression was also reportedly affected in those patients 43. In the current study, TGFβ1 expression in ocular fibroblasts was suppressed by the loss of Tnx gene function. Reduction of TGFβ1 expression in mesenchymal cells might be attributable to the inhibition of the early‐phase invasion of macrophages in the stroma. TGFβ1 expression exhibits a positive feedback effect, and activation of TGFβ is considered to be followed by augmentation of TGFβ1expression. The exact mechanism of inhibition of TGFβ expression by the loss of Tnx gene function remains to be clarified; our data using Western blotting showed that loss of Tnx gene function does not affect Smad3 phosphorylation in ocular fibroblasts (data not shown). Nevertheless, another group reported that TNX activates a latent form of endogenous TGFβ1 and accelerates epithelial‐mesenchymal transition in cell culture, which is in agreement with the current data 34. Our preliminary experiments clearly showed that mouse macrophages express a significant level of *Tnx* mRNA (data not shown). Thus, we also examined if lacking Tnx affects VEGF expression in macrophages and showed that VEGF‐A mRNA expression was markedly suppressed in KO macrophages as compared with WT macrophages. We therefore propose that both reduction of macrophage infiltration in KO tissues and decreased expression of VEGF‐A in KO macrophages account for why declines in tissue VEGF‐A level inhibited stromal neovascularization in a KO mouse.

In conclusion, endogenous Tnx modulates expression of both VEGF‐A and ‐B and is involved in development of corneal neovascularization. It is relevant to obtain a better understanding of how angiogenic factor expression is modulated by matricellular proteins because it can lead to the development of new strategies to overcome sight compromising corneal neovascularization.

## Conflict of interest

There is no conflict of interest.
